# Rapid diagnosis of intra-amniotic infection using nanopore-based sequencing

**DOI:** 10.1515/jpm-2022-0504

**Published:** 2023-07-26

**Authors:** Piya Chaemsaithong, Roberto Romero, Pisut Pongchaikul, Pornpun Vivithanaporn, Waranyu Lertrut, Adithep Jaovisidha, Paninee Mongkolsuk, Perapon Nitayanon, Khontawan Pongsuktavorn, Threebhorn Kamlungkuea, Eunjung Jung, Manaphat Suksai, Arunee Singhsnaeh, Piroon Jenjaroenpun, Iyarit Thaipisuttikul, Thidathip Wongsurawat

**Affiliations:** Department of Obstetrics and Gynecology, Faculty of Medicine Ramathibodi Hospital, Mahidol University, Bangkok, Thailand; Department of Obstetrics and Gynecology, Wayne State University School of Medicine, Detroit, MI, USA; Perinatalogy Research Branch, Division of Obstetrics and Maternal-Fetal Medicine, Division of Intramural Research, Eunice Kennedy Shriver National Institute of Child Health and Human Development, National Institutes of Health, U. S. Department of Health and Human Services, Bethesda, MD, and Detroit, MI, USA; National Institutes of Health, Bethesda, MD, USA; Department of Obstetrics and Gynecology, University of Michigan, Ann Arbor, MI, USA; Department of Epidemiology and Biostatistics, Michigan State University, East Lansing, MI, USA; Center for Molecular Medicine and Genetics, Wayne State University, Detroit, MI, USA; Detroit Medical Center, Detroit, MI, USA; Faculty of Medicine Ramathibodi Hospital, Chakri Naruebodindra Medical Institute, Mahidol University, Samut Prakarn, Thailand; Integrative Computational Bioscience (ICBS) Center, Mahidol University, Nakorn Pathom, Thailand; Institute of Infection, Veterinary and Ecological Sciences, University of Liverpool, Liverpool, UK; Department of Microbiology, Faculty of Medicine Siriraj Hospital, Mahidol University, Bangkok, Thailand; Siriraj Genomics, Molecular Genetics Laboratory, Office of the Dean, Faculty of Medicine Siriraj Hospital, Mahidol University, Bangkok, Thailand; Department of Pathology, Faculty of Medicine, Ramathibodi Hospital, Mahidol University, Bangkok Thailand; Division of Medical Bioinformatics, Department of Research, Faculty of Medicine Siriraj Hospital, Mahidol University, Bangkok, Thailand; Siriraj Long-Read Lab (Si-LoL), Department of Research, Faculty of Medicine Siriraj Hospital, Mahidol University, Bangkok, Thailand

**Keywords:** 16S rDNA, amniocentesis, amniotic fluid, bacteria, infection, intra-amniotic inflammation, long-read sequencing, microorganism, preterm labor, Sanger sequencing

## Abstract

**Objectives:**

Early diagnosis and treatment of intra-amniotic infection is crucial. Rapid pathogen identification allows for a definite diagnosis and enables proper management. We determined whether the 16S amplicon sequencing performed by a nanopore sequencing technique make possible rapid bacterial identification at the species level in intra-amniotic infection.

**Methods:**

Five cases of confirmed intra-amniotic infection, determined by either cultivation or 16S rDNA polymerase chain reaction (PCR) Sanger sequencing, and 10 cases of women who underwent mid-trimester genetic amniocentesis were included. DNA was extracted from amniotic fluid and PCR was performed on the full-length 16S rDNA. Nanopore sequencing was performed. The results derived from nanopore sequencing were compared with those derived from cultivation and Sanger sequencing methods.

**Results:**

Bacteria were successfully detected from amniotic fluid using nanopore sequencing in all cases of intra-amniotic infection. Nanopore sequencing identified additional bacterial species and polymicrobial infections. All patients who underwent a mid-trimester amniocentesis had negative cultures, negative 16S PCR Sanger sequencing and nanopore sequencing. Identification of the microorganisms using nanopore sequencing technique at the bacterial species level was achieved within 5–9 h from DNA extraction.

**Conclusions:**

This is the first study demonstrating that the nanopore sequencing technique is capable of rapid diagnosis of intra-amniotic infection using fresh amniotic fluid samples.

## Introduction

The amniotic cavity is normally sterile, and microbial invasion elicits an inflammatory response in patients with spontaneous preterm labor [1], cervical insufficiency [2], and clinical chorioamnionitis at term [3], among other obstetrical syndromes. Intra-amniotic inflammation is defined by an elevation of the interleukin-6 (IL-6) concentration (IL-6 ≥2.6 ng/mL), and intra-amniotic infection is a combination of demonstrable microorganisms in the amniotic cavity and intra-amniotic inflammation [1]. The accurate diagnosis of infection requires identification of microorganisms from samples obtained from the suspected infection site, and in clinical medicine, this is accomplished with cultivation techniques, which have remained the gold standard for microbial identification for more than 100 years [4]. However, the result of a culture takes days to become available, and this delay has been a major obstacle for the successful and timely treatment of intra-amniotic infection in obstetrics and other infections, virtually in every field of medicine. Molecular microbiologic techniques promise to address this problem by analysis of nucleic acids of microorganisms. The most widely used approach is the combination of the polymerase chain reaction (PCR) of the conserved region of the microbial genomes, also known as 16S rDNA PCR (16S ribosomal DNA), and of sequencing of the amplicon to identify genus and specificity. However, this technique generally takes days. Nanopore sequencing has recently emerged as a unique scalable method that enables direct real-time analysis of long DNA fragments, thus making possible rapid identification of bacteria, viruses, or fungi with great specificity [5, 6]. This technology has been successfully used to diagnose infection based on sequencing of organisms grown in culture, pus, cerebrospinal fluid, blood, as well as prosthetic devices [7]. The present study was conducted to determine whether nanopore sequencing can identify bacteria directly from amniotic fluid of patients suspected to have intra-amniotic infection.

## Materials and methods

Nanopore sequencing of DNA obtained from amniotic fluid samples of cases and controls was performed. The control group comprised patients who underwent genetic amniocentesis in the mid-trimester and who had a negative amniotic fluid culture and negative 16S rDNA PCR Sanger sequencing, as well as a delivery at term (n=10). Cases consisted of patients with preterm pre-labor ruptured of membranes (preterm PROM) who underwent transabdominal amniocentesis to determine the microbial state in the amniotic cavity and were found to have microorganisms by either cultivation or 16S rDNA PCR Sanger sequencing (n=5). Each patient provided written informed consent, and the use of biological specimens and clinical data for research purposes was approved by Institutional Review Boards of Faculty of Medicine Ramathibodi Hospital, Mahidol University (COA.MURA2021/254 and COA.MURA2021/968).

Background technical controls included DNA extractions performed on (1) DNA extraction kits without amniotic fluid samples; (2) extraction kits with bead tubes exposed to room air for 20 min during amniocentesis; (3) extraction kits with bead tubes exposed to conditions similar to those of amniotic fluid samples undergoing testing (i.e., alcohol, betadine, and container). Amniotic fluid IL-6 concentrations (ng/mL) were determined by enzyme-linked immunosorbent assay (ELISA; R&D Systems, Minneapolis, MN, USA). The details and performance of the ELISAs were previously described [3]. Intra-amniotic inflammation was considered to be present if the amniotic fluid IL-6 concentration was ≥2.6 ng/mL [3]. Intra-amniotic infection was defined as the presence of intra-amniotic inflammation with demonstrable microorganism in the amniotic cavity [1].

### Detection of bacteria by the 16S rDNA PCR Sanger sequencing method

Amniotic fluid was centrifuged at 5,000×*g*, 4 °C for 10 min. Amniotic fluid supernatant was collected for IL-6 determination. The genomic DNA was extracted from 1 mL of fresh amniotic fluid using the ZymoBIOMICS kit (Zymo Research Corporation, Irvine, CA, USA) and then the genomic DNA was used as a template for amplifying full-length 16S rDNA genes. 16s rDNA PCR was performed by using 27F and 1492R primers with 35 cycles. DNA sequencing of the amplicon was determined by the Sanger method [8]. Raw DNA sequences were manually visualized and curated in BioEdit [9, 10]. Bacterial identification was performed by searching the curated DNA sequences via nucleotide BLAST [11].

### Nanopore sequencing technique

The 16S Barcoding Kit (SQK-RAB204; Oxford Nanopore Technologies, Oxford, United Kingdom) [12] was used for DNA library preparation. PCR amplification was conducted with LongAmp™ Taq 2× Master Mix (New England Biolabs, Ipswich, MA, USA). Amplification was performed under the following conditions: initial denaturation at 95 °C for 1 min, 25 cycles of 95 °C for 20 s, 55 °C for 30 s, and 65 °C for 2 min, followed by a final extension at 65 °C for 5 min. The PCR product of each sample was cleaned up and concentrated with AMPure XP (Beckman Coulter, Indianapolis, IN, USA). A total of 10 μL purified DNA was used for library preparation. MinION Mk1C sequencing was performed by using R9.4.1 flow cells (ONT) [5, 13].

### Bioinformatics analysis

The nanopore raw data (fast5 files) were base-called and de-multiplexed with ONT’s Guppy™ software version 6.2.1 “super accuracy” model (-c dna_r9.4.1_450 bps_sup.cfg). The adapter sequences were trimmed by using Porechop software v.2.6.0 (https://github.com/rrwick/Porechop). During data preprocessing, reads were filtered with NanoFilt to retain only near full-length 16S rDNA reads. Reads with a length below 1,000 base pairs (bp) or a Q-score below 9 were discarded. Fastq 16S workflows in the cloud-based bioinformatics platform EPI2ME identified the pathogens.

## Results

The clinical characteristics of the patients are shown in Table 1. DNA samples of the background technical controls had negative cultures, negative 16S rDNA PCR Sanger sequencing and negative nanopore sequencing results. All patients in the control group (mid-trimester amniocentesis with a negative amniotic fluid culture and no evidence of microorganisms determined by 16S rDNA Sanger sequencing) had no bacteria detected by nanopore sequencing. On the other hand, using nanopore sequencing, bacterial nucleic acid was detected in all 5 patients diagnosed with intra-amniotic infection (either by cultivation or 16S rDNA Sanger sequencing). All bacterial genera identified by culture or Sanger sequencing were also detected by nanopore sequencing; however, nanopore sequencing identified additional species of bacteria in 3 of the 5 cases (case #1, #2 and #4) (Table 1).

**Table 1:** Clinical characteristics of patients with intra-amniotic infection.

Patient number	Diagnosis	Gestational age at amniocentesis, weeks of gestation	Gestational age at delivery, weeks of gestation	Maternal WBC, /mm^3^ and C-reactive protein, mg/dL	Amniotic fluid interleukin-6, ng/mL	Amniotic fluid culture result	16S rDNA PCR Sanger sequencing result	Nanopore sequencing result	Placental histopathology
1	Preterm PROM	31^+2^	31^+3^	18,890 and 22.92	43.6	*Streptococcus anginosus*	*Streptococcus anginosus*	*Streptococcus vaginalis* *Streptococcus intermedius* *Streptococcus constellatus*	Acute chorioamnionitis stage 2 grade 2
2	Preterm PROM	32	32	22,760 and 7.95	19.6	*Streptococcus mitis*	Unknown	*Streptococcus mitis* *Streptococcus oralis* *Peptoniphilus spp.* *Prevotella bivia*	Acute chorioamnionitis stage 2 grade 2
3	Preterm PROM	21	21^+3^	15,400 and 76.4	103.6	No growth	*Ureaplasma urealyticum*	*Ureaplasma urealyticum*	Acute chorioamnionitis stage 2 grade 2
4	Preterm PROM	32^+1^	33^+5^	9,970 and 6.72	8.2	No growth	*Veillonella spp.*	*Veillonella montpellierensis* *Ureaplasma parvum* *Veillonella atypica*	Acute chorioamnionitis stage 2 grade 2
5	Preterm PROM	27^+4^	27^+4^	16,530 and 70	18.2	*Gardnerella vaginalis*	*Gardnerella vaginalis*	*Gardnerella vaginalis*	Acute chorioamnionitis stage 2 grade 2

16S rDNA PCR, 16S rDNA-based polymerase chain reaction; PROM, prelabor ruptured of membranes; WBC, white blood cell count. Intra-amniotic inflammation is defined as an amniotic fluid interleukin-6 concentration ≥2.6 ng/mL.

None of the patients had evidence of clinical chorioamnionitis and all patients with intra-amniotic infection had intra-amniotic inflammation, as well as acute histologic chorioamnionitis. In 2 cases (#3 and #5), there was concordance among the results of either culture or 16S rDNA PCR Sanger sequencing, and nanopore sequencing. In case #1, the culture and 16S rDNA PCR Sanger sequencing yielded *Streptococcus anginosus*, but nanopore sequencing identified 3 additional species: *Streptococcus vaginalis, Streptococcus intermedius* and *Streptococcus constellatus.* In case #4, culture of amniotic fluid was negative; however, 16S rDNA PCR Sanger sequencing identified *Veillonella* spp., and nanopore sequencing identified *Veillonella montpellierensis*, *Veillonella atypica*, and *Ureaplasma parvum*, which were not identified by 16S rDNA PCR Sanger sequencing or by culture.

## Discussion

This is the first report of the successful use of nanopore sequencing to diagnose intra-amniotic infection. A key observation is that nanopore sequencing detected bacteria by analyzing a fresh biological sample, e.g., amniotic fluid rather than material obtained from culture. One study has reported the identification of bacteria in cerebrospinal fluid in cases of suspected meningitis [14]. Other biological specimens in which nanopore sequencing has been used for the same purpose include whole blood, plasma, serum, sputum, feces, and sonicated fluid obtained from a prosthetic joint infection [7].

Molecular sequencing-based microbial identification has allowed to improve the accuracy of diagnosis of infection by enhancing the identification of non-culturable organisms [15]. The 16S rDNA-based PCR assay, that amplifies phylogenetically informative regions of the 16S rDNA gene sequence, identifies microbes across broad taxonomic fields, including previously uncharacterized species [15]. Several generations of gene sequencing techniques have emerged over time. The most widely used is 16S amplicon sequencing by short-read technology is less optimal than long-read technology in the identification of bacteria at the species level.

**Figure 1:**
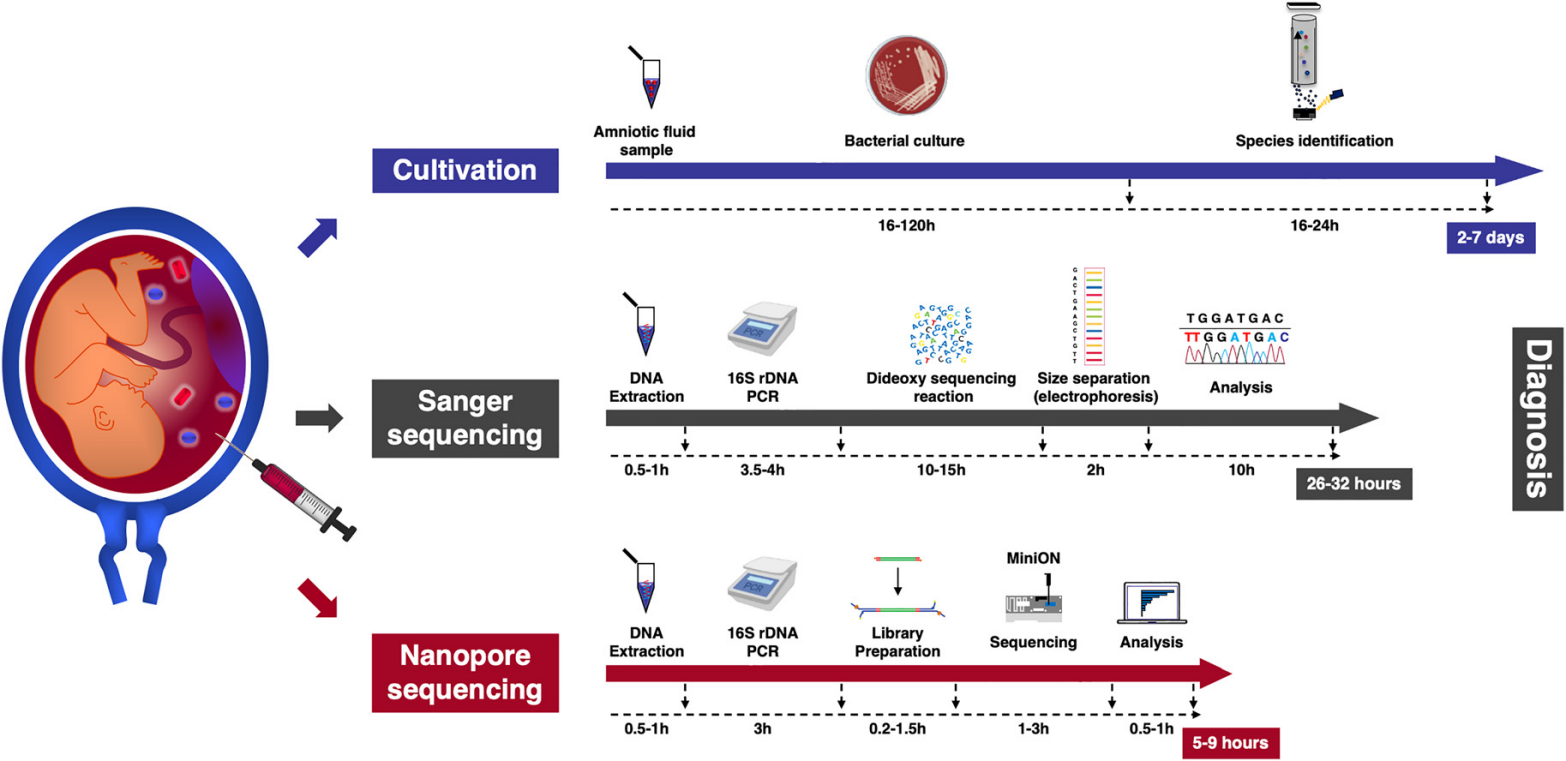
Estimated turnaround time of each diagnostic method. The conventional bacterial cultivation usually requires 2–7 days to identify the bacteria. The 16S Sanger sequencing method usually lasts 26–32 h. The time analysis of Sanger sequencing is based on Shadi Shokralla et al. [17]. However, the time-to-result of Sanger sequencing depends on the number of samples. 16S nanopore sequencing can provide the result for the identification of intra-amniotic infection within 9 h from DNA extraction. Indeed, the nanopore and Sanger sequencing methods require approximately 3 h for 16S rRNA PCR amplification. However, the library preparation (dideoxy sequencing reaction) of 16S Sanger sequencing takes considerably longer (∼10–15 h) compared to 16S nanopore sequencing. For nanopore sequencing, the library preparation takes approximately 0.2–1.5 h as the clean PCR amplicon can be immediately attached to the rapid adaptor. Then, the library can be loaded and the analysis can be performed in real-time, which may take up to 3 h to obtain the result.

Nanopore sequencing, a third-generation sequencing technique, makes possible real-time analysis. Long read technology (up to several million base pairs) allows identification of microorganisms at the species level [6, 16]. The advantages of nanopore sequencing are two-fold: the technique is faster and more sensitive than culture and it can be used when patients have been treated with antibiotics. Due to the fastidious nature of microorganisms causing intra-amniotic infection, the standard culture may take 2–7 days while nanopore sequencing identifies bacteria within 5–9 h from DNA extraction (Figure 1) [14, 17]. In the present study, nanopore sequencing identified bacterial species within 4.5 h after the DNA extraction process. However, the time required to generate results depends on the microbial burden. Importantly, nanopore sequencing can detect polymicrobial infections, which can be a challenge when using 16S rDNA PCR Sanger sequencing [5, 6]. The accessibility and portability of this sequencing technique are features that promise a timely point-of-care test to detect intra-amniotic infection in the clinical setting in order to improve the clinical care of mothers and newborns. Knowledge of the presence of bacteria and the specific species can accelerate decision-making to deliver or to administer a particular antibiotic to treat a specific microorganism. In practice, a rapid IL-6 or a rapid bedside MMP-8 test of amniotic fluid can be used for the rapid diagnosis of intra-amniotic inflammation [18], [19], [20], [21] and nanopore sequencing would allow the diagnosis of the causative microbe or, in the absence of microorganisms, sterile intra-amniotic inflammation. The knowledge of the specific microorganism could be particularly helpful to the neonatologist to select the antimicrobial agents appropriate for each particular newborn. For example, the identification of candida or genital mycoplasmas would require antibiotics not used as part of standard of care in newborn intensive care units. Further studies are needed to confirm our findings and to optimize nanopore sequencing methods for amniotic fluid samples, targeted organisms, the amount of nucleic acid isolated, the sequencing approach, and the preparation of a proper genomic DNA library.

## Conclusions

Nanopore sequencing technology is a promising method to detect intra-amniotic infection, given rapid library preparation, real-time sequencing, and simplicity. This method represents an improvement over current molecular microbiologic techniques by allowing cataloging the organisms present within complex polymicrobial bacterial communities, directly from patient specimens. The real-time technology is ideal for detecting and identifying pathogens in patients in labor and delivery units, as timing is crucial when initiating antibiotic treatment or delivery. This report is the first to demonstrate that this technique can identify bacteria from a fresh sample of amniotic fluid within 9 h from DNA extraction. In addition, nanopore sequencing is superior to traditional cultivation and 16S rDNA Sanger sequencing in several aspects, i.e., its ability to detect polymicrobial infections and non-culturable bacteria.
